# *cyclo*-Tetra­kis­(μ-2,4,6-tri­methyl­phenyl-κ*C* ^1^:κ*C* ^1^)bis­(tri­methyl­phosphane)-1κ*P*,3κ*P*-tetra­copper(I)

**DOI:** 10.1107/S2414314621005940

**Published:** 2021-06-15

**Authors:** Phil Liebing, Kurt Merzweiler

**Affiliations:** a Martin-Luther-Universität Halle, Naturwissenschaftliche Fakultät II, Institut für Chemie, Germany; Vienna University of Technology, Austria

**Keywords:** crystal structure, copper, phosphine, complex

## Abstract

The title compound, [Cu_4_(C_9_H_11_)_4_(C_3_H_9_P)_2_] or [Cu_4_(Mes)_4_(PMe_3_)_2_] (Mes = 2,4,6-tri­methyl­phenyl), was synthesized from copper(I) mesityl and tri­methyl­phosphane in THF as solvent. The mol­ecular structure of the complex has *C*_2_ symmetry and consists of four copper(I) atoms bridged by four *μ*-mesityl groups, giving an eight-membered puckered {Cu_4_C_4_} ring. Additionally, two copper(I) atoms at opposite corners of the Cu_4_ rhomb are each linked to a terminal PMe_3_ ligand. The PMe_3_-bearing copper(I) atoms exhibit a distorted trigonal–planar coordination mode whereas the remaining Cu atoms linked to two mesityl groups are nearly linearly coordinated.

## Abstract 2

The mol­ecular structure of the title complex consists of an eight-membered Cu_4_C_4_ ring with an alternating arrangement of copper(I) atoms and *μ*-mesityl groups. Two of the copper(I) atoms are additionally linked to PMe_3_ ligands, giving a distorted trigonal coordination.

## Graphical abstract

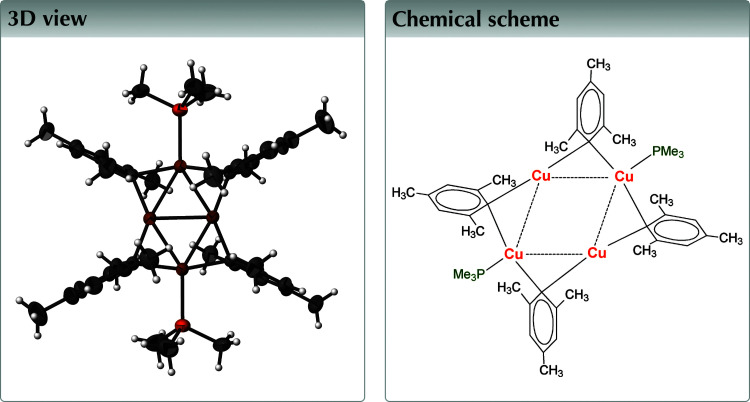

## Structure description

Among Cu^I^ organyls, mesitylcopper is one of the most extensively studied compounds. Since its first synthesis in 1981 (Tsuda *et al.*, 1981 ▸), mesitylcopper has found widespread application in preparative organometallic chemistry (Stollenz & Meyer, 2012 ▸). In the solid state, mesitylcopper can exist as a penta­mer (CuMes)_5_ (Gambarotta *et al.*, 1983 ▸; Meyer *et al.*, 1989 ▸) or as a tetra­mer (CuMes)_4_ (Eriksson & Håkansson, 1997 ▸). On treatment with donor ligands *L*, mesitylcopper displays different reaction patterns depending on the nature of *L*. In the case of tetra­hydro­thio­phene (THT), the reaction proceeds under retention of the tetra­nuclear cluster structure to form [Cu_4_(Mes)_4_(THT)_2_] (Gambarotta *et al.*, 1983 ▸; Meyer *et al.*, 1989 ▸). Treatment of mesitylcopper with PPh_3_ in toluene led to a compound [CuMes(PPh_3_)_2_]·C_7_H_8_ with a yet unknown crystal structure (Meyer *et al.*, 1989 ▸). The reaction with dppe (1,2-bis­(di­phenyl­posphino)ethane) causes a degradation of the Cu_4_Mes_4_ cluster to give a cuprocuprate [(dppe)_2_Cu][CuMes_2_] (Leoni *et al.*, 1983 ▸).

In order to get some insight into the reactivity of mesitylcopper towards sterically less demanding phosphanes, tri­methyl­phosphane was chosen as a ligand. Treatment of a solution of mesitylcopper in THF with PMe_3_ at room temperature led to the formation of the tetra­nuclear complex [Cu_4_(Mes)_4_(PMe_3_)_2_] (**1**).

**Figure 1:**
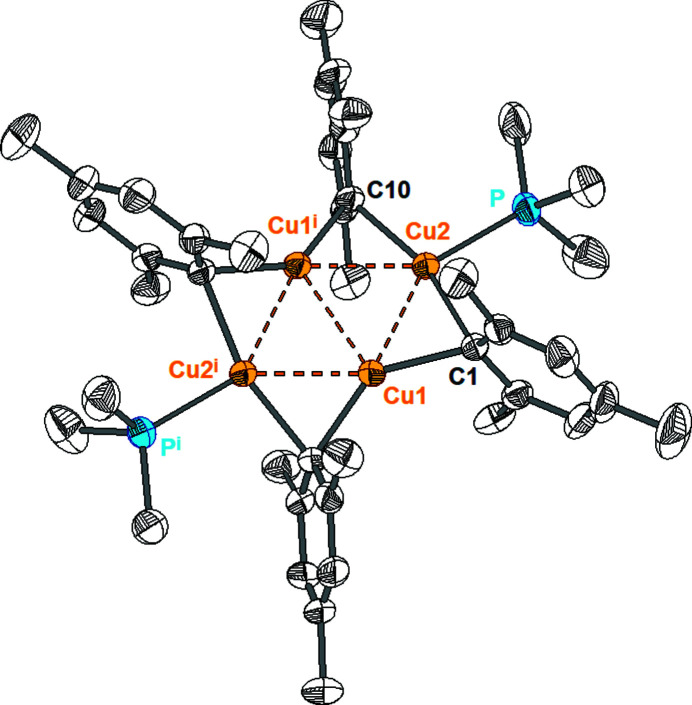
Mol­ecular structure of [Cu_4_Mes_4_(PMe_3_)_4_] showing the labeling scheme. Displacement ellipsoids were drawn at the 50% probability level, H atoms are omitted for clarity. [Symmetry code: (i) −*x* + 1, *y*, −*z* + 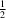 ].

The mol­ecular structure of (**1**) comprises four copper(I) atoms that are linked by four *μ*-mesityl groups to give an eight-membered {Cu_4_C_4_} ring (Fig. 1 ▸). Additionally, two copper atoms at diametrically opposite positions of the ring are each linked to a terminal PMe_3_ group. The tetra­nuclear copper complex exhibits crystallographic *C*_2_ symmetry with the diad axis passing through the center of the C10—C15 bond. The rhombic arrangement of the copper atoms is nearly planar, with marginal deviations of 0.0087 Å from the mean plane through the four copper atoms. The relatively small Cu⋯Cu distances at the edges of the rhomb [2.4603 (5)–2.4625 (5) Å] suggest cuprophilic inter­actions. The Cu⋯Cu separations between the copper atoms at opposite corners of the rhomb are 4.2013 (5) Å for Cu2⋯Cu2^i^ [symmetry code: (i) –*x* + 1, *y*, –*z* + 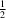 ] and 2.5657 (7) Å for Cu1⋯Cu1^i^. Similar shaped arrangements of four Cu atoms were observed in the derivatives [Cu_4_(Mes)_4_(THT)_2_], [Cu_4_(*o*-Tol)_4_(SMe_2_)_2_] (Lend­ers *et al.*, 1991 ▸) and [Cu_4_Ph_4_(SMe_2_)_2_] (Olmstead & Power, 1990 ▸). Complex (**1**) exhibits two types of differently coordin­ated Cu atoms (Table 1 ▸). Cu1 is surrounded by two mesityl groups with Cu—C distances of 2.005 (3) and 2.006 (3) Å. In comparison with Cu_4_Mes_4_, the Cu—C distances are slightly enlarged by around 0.014 Å. However, the bending of the C1—Cu1—C10^i^ unit [138.3 (1)°] is clearly more pronounced than in [Cu_4_Mes_4_] (164.05–165.70°). Apart from two mesityl groups, Cu2 bears a PMe_3_ unit as a third ligand. The increased coordination number leads to a further enlargement of the Cu—C distances with values of 2.093 (3) and 2.095 (3) Å. The coordination around Cu2 is planar with a C—Cu—C angle of 163.0 (1)° and C—Cu—P angles of 97.9 (1)° and 99.0 (1)° (sum of the angles around Cu2: 359.9°). Comparison of the bond lengths of compound (**1**) and related [Cu_4_Mes_4_ *L*_2_] complexes reveals that the ligand PMe_3_ leads to a larger increase of the Cu—C distances for the tricoordinate copper atoms than other ligands investigated so far. In [Cu_4_Mes_4_ *L*_2_] complexes with *L* = piperidine, allyl methyl sulfide, 2,5-di­thia­hexane, tetra­hydro­thio­phene and bis­{2-[1-(di­methyl­amino)­eth­yl]phenyl­thiol­ato}magnesium, the mean Cu—C distances for the tricoordinated copper atoms are in the range 2.054–2.064 Å. In the case of the dicoordinated Cu there is no particular effect. Furthermore, there is a slight influence on the C—Cu—C angles for the dicoordinated [138.3 (1)°] and the tricoordinate copper atoms [163.0 (1)°], which are smaller than in the [Cu_4_Mes_4_ *L*_2_] complexes mentioned above (140.3–142.8° and 165.0–170.2°, respectively).

**Figure 2:**
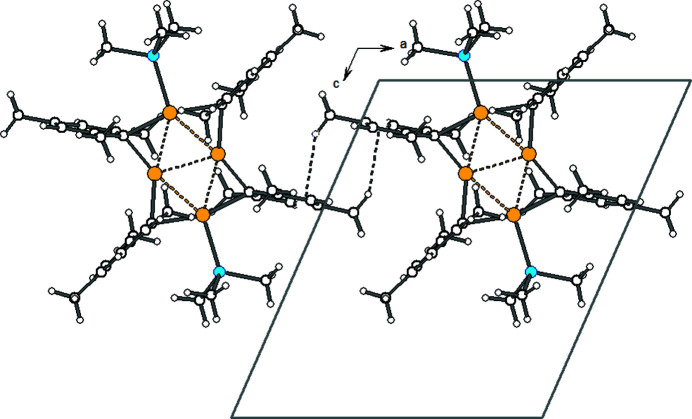
Partial packing diagram for **1** in a view down the crystallographic *b* axis. The inter­molecular C—H⋯π inter­actions are shown as gray dashed lines.

The mol­ecular packing reveals no special supra­molecular features (Fig. 2 ▸). Most of the contacts are of the van der Waals type with some minor participation of C—H⋯*π* inter­actions: C17—H17*A*⋯*Cg*2^i^ with *d*(H⋯*Cg*2) = 2.87 Å, C17—H17*A*⋯*Cg*2 = 167° [*Cg*2 is the centroid of the C10–C15 ring; symmetry code: (i) −x, *y*, 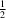 − *z*].

Generally, X-ray crystallographic studies on Cu^I^ aryl compounds with auxiliary phosphane ligands are relatively rare. According to the CSD database (Groom *et al.*, 2016 ▸), there are two compounds of the type [*R*Cu(P*R*′_3_)] [*R* = (2,2′′,4,4′′,6,6′′-hexa­methyl-1,1′:3′,1′′-terphenyl-2′-yl), *R*′ = Ph (Niemeyer, 2003 ▸), *R*′= Et (Rungthanaphatsophon *et al.*, 2016 ▸], containing nearly linear C—Cu—P units. Typically, this structural motif occurs if further mol­ecular aggregation is prevented by sterically demanding aryl groups. The Cu—C bond lengths in [*R*Cu(P*R*′_3_)]-type compounds are 1.922 Å for the PPh_3_ derivative (Niemeyer, 2003 ▸) and 1.930 Å in the case of the PEt_3_ co-ligand. The shortening of the Cu—C distances in comparison with [Cu_4_(Mes)_4_(PMe_3_)_2_] may be attributed to the lower coordination number of the copper atoms. The same effect is also visible for the Cu—P distances of 2.189 Å (Niemeyer, 2003 ▸) and 2.200 Å (Rungthanaphatsophon *et al.*, 2016 ▸), respectively. Furthermore, there is a terphenyl copper complex of the type [*R*Cu(P*R*′_3_)_2_] (*R*′= Et) with two phosphane units attached to copper. In this case, the copper atom exhibits a distorted trigonal–planar coordination with markedly enlarged Cu—C (1.979 Å) and Cu—P (2.250 and 2.256 Å) distances (Rungthanaphatsophon *et al.*, 2016 ▸).

The CSD database contains five entries for [Cu_4_Mes_4_ *L*_2_] complexes, with *L* = THT (Gambarotta *et al.*, 1983 ▸; Meyer, *et al.*, 1989 ▸), piperidine (Sung *et al.*, 2015 ▸), allyl methyl sulfide or 2,5–di­thia­hexane (Kokoli *et al.*, 2013 ▸). There are also some heterometallic Cu_4_Mes_4_ complexes with bis­(thio­pheno­lato)magnesium units as ligands (Knotter *et al.*, 1990 ▸).

## Synthesis and crystallization

A solution of 0.46 g (2.5 mmol) mesitylcopper (Meyer *et al.*, 1989 ▸) in 10 ml of THF was treated with 0.13 ml (1.25 mmol) of trimethyl phosphane. The reaction mixture was stirred for one h at 293 K. The reaction product [Cu_4_(Mes)_4_(PMe_3_)_2_] (**1**) was precipitated by the addition of 30 ml of *n*-hexane. After filtration, the colorless product was washed with diethyl ether (2 × 5 ml) and dried under vacuum. Single crystals suitable for X-ray analysis were obtained by slow diffusion of *n*-hexane into a THF solution of the product. Yield: 0.33 g (60%). C_42_H_62_Cu_4_P_2_ (883.01 g mol^−1^). Analysis: Cu 29.0% (calc. 28.8%) IR (cm^−1^) 2997(*w*), 2963(*m*), 2901(*m*), 2855(*w*), 2842(*w*), 2802(*w*), 2705(*w*), 1589(*w*), 1637(*w*), 1376(*w*), 1448(*w*), 1418(*m*), 1362(*w*), 1302(*w*), 1286(*m*), 1257(*w*), 1215(*w*), 1164(*w*), 1024(*w*), 939(*s*), 873(*w*), 844(*s*), 730(*s*), 710(*w*), 670(*m*), 576(*w*), 538(*m*), 484(*w*), 357(*m*), 328(*m*), 301(*m*), 275(*w*). ^1^H NMR (C_6_D_6_): δ 0.60 (*s br*, 18H; PCH_3_), 2.08 (*s*, 12H; *p*-CH_3_), 2.73 (*s*, 24H; *o*-CH_3_), 6.68 (*s*, 8H; CH). ^13^C{^1^H} NMR (C_6_D_6_): δ 15.4 (*s br*; PCH_3_), 21.4 (*s*; *p*-CH_3_), 28.7 (*s*; *o*-CH_3_), 125.6 (*s*; CH), 127.4 (*s*; CCu_2_), 134.4 (*s*; *p*-CCH_3_), 150.0 (*s*; *o*-CCH_3_). ^31^P{^1^H} NMR (C_6_D_6_): δ −44.6 (*s br*).

## Refinement

Crystal data, data collection and structure refinement details are summarized in Table 2 ▸.

## Supplementary Material

Crystal structure: contains datablock(s) I. DOI: 10.1107/S2414314621005940/wm4146sup1.cif

Structure factors: contains datablock(s) I. DOI: 10.1107/S2414314621005940/wm4146Isup2.hkl

CCDC reference: 2088681

Additional supporting information:  crystallographic information; 3D view; checkCIF report

## Figures and Tables

**Table 1:** Selected geometric parameters (Å, °)

C1—Cu1	2.006 (3)	P—Cu2	2.2967 (9)
C1—Cu2	2.095 (3)	Cu1—Cu1^i^	2.5657 (7)
C10—Cu1^i^	2.005 (3)	Cu1—Cu2	2.4603 (5)
C10—Cu2	2.093 (3)	Cu1—Cu2^i^	2.4625 (5)
			
C10^i^—Cu1—C1	138.26 (11)	C10—Cu2—C1	163.04 (11)
C1—Cu2—P	97.94 (8)	C10—Cu2—P	99.02 (8)

Symmetry code: (i) 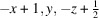 .

**Table 2:** Experimental details

Crystal data	
Chemical formula	[Cu_4_(C_9_H_11_)_4_(C_3_H_9_P)_2_]
*M*_r_	883.01
Crystal system, space group	Monoclinic, *C*2/*c*
Temperature (K)	213
*a*, *b*, *c* (Å)	12.0750 (8), 27.5202 (18), 14.3164 (9)
β (°)	113.668 (5)
*V* (Å^3^)	4357.3 (5)
*Z*	4
Radiation type	Mo *K*α
μ (mm^−1^)	2.03
Crystal size (mm)	0.51 × 0.32 × 0.19

Data collection	
Diffractometer	Stoe IPDS 2
Absorption correction	Integration (*X-AREA*; Stoe & Cie, 2016 ▸)
*T*_min_, *T*_max_	0.508, 0.758
No. of measured, independent and observed [*I* > 2σ(*I*)] reflections	10982, 3839, 3043
*R*_int_	0.050
(sin θ/λ)_max_ (Å^−1^)	0.597

Refinement	
*R*[*F* ^2^ > 2σ(*F* ^2^)], *wR*(*F* ^2^), *S*	0.033, 0.100, 1.04
No. of reflections	3839
No. of parameters	217
H-atom treatment	H-atom parameters constrained
Δρ_max_, Δρ_min_ (e Å^−3^)	0.48, −0.44

Computer programs: (*X-AREA*; Stoe & Cie, 2016 ▸), *SHELXT* (Sheldrick, 2015*a* ▸), *SHELXL* (Sheldrick, 2015*b* ▸) and *OLEX2* (Dolomanov *et al.*, 2009 ▸).

## full crystallographic data

### Crystal data

**Table:**

[Cu_4_(C_9_H_11_)_4_(C_3_H_9_P)_2_]	*F*(000) = 1840
*M**_r_* = 883.01	*D*_x_ = 1.346 Mg m^−^^3^
Monoclinic, *C*2/*c*	Mo *K*α radiation, λ = 0.71073 Å
*a* = 12.0750 (8) Å	Cell parameters from 10871 reflections
*b* = 27.5202 (18) Å	θ = 1.5–25.6°
*c* = 14.3164 (9) Å	µ = 2.03 mm^−^^1^
β = 113.668 (5)°	*T* = 213 K
*V* = 4357.3 (5) Å^3^	Block, pale yellow
*Z* = 4	0.51 × 0.32 × 0.19 mm

### Data collection

**Table:**

Stoe IPDS 2 diffractometer	3043 reflections with *I* > 2σ(*I*)
rotation method scans	*R*_int_ = 0.050
Absorption correction: integration (X-AREA; Stoe & Cie, 2016)	θ_max_ = 25.1°, θ_min_ = 1.5°
*T*_min_ = 0.508, *T*_max_ = 0.758	*h* = −14→13
10982 measured reflections	*k* = −32→31
3839 independent reflections	*l* = −17→17

### Refinement

**Table:**

Refinement on *F*^2^	0 restraints
Least-squares matrix: full	Hydrogen site location: inferred from neighbouring sites
*R*[*F*^2^ > 2σ(*F*^2^)] = 0.033	H-atom parameters constrained
*wR*(*F*^2^) = 0.100	*w* = 1/[σ^2^(*F*_o_^2^) + (0.0519*P*)^2^ + 5.6305*P*] where *P* = (*F*_o_^2^ + 2*F*_c_^2^)/3
*S* = 1.03	(Δ/σ)_max_ < 0.001
3839 reflections	Δρ_max_ = 0.48 e Å^−^^3^
217 parameters	Δρ_min_ = −0.44 e Å^−^^3^

### Special details

**Table:**

Geometry. All esds (except the esd in the dihedral angle between two l.s. planes) are estimated using the full covariance matrix. The cell esds are taken into account individually in the estimation of esds in distances, angles and torsion angles; correlations between esds in cell parameters are only used when they are defined by crystal symmetry. An approximate (isotropic) treatment of cell esds is used for estimating esds involving l.s. planes.

### Fractional atomic coordinates and isotropic or equivalent isotropic displacement parameters (Å^2^)

**Table:**

	*x*	*y*	*z*	*U*_iso_*/*U*_eq_	
C1	0.5285 (3)	0.14472 (10)	0.0734 (2)	0.0352 (6)	
C2	0.5698 (3)	0.11189 (11)	0.0184 (2)	0.0406 (7)	
C3	0.6133 (3)	0.12859 (13)	−0.0520 (3)	0.0535 (9)	
H3	0.637972	0.106131	−0.088230	0.064*	
C4	0.6210 (3)	0.17740 (14)	−0.0697 (3)	0.0567 (9)	
C5	0.5825 (3)	0.21013 (13)	−0.0155 (3)	0.0542 (9)	
H5	0.587281	0.243244	−0.026399	0.065*	
C6	0.5369 (3)	0.19478 (11)	0.0549 (2)	0.0422 (7)	
C7	0.5670 (3)	0.05827 (11)	0.0365 (3)	0.0530 (8)	
H7A	0.534717	0.052614	0.086869	0.064*	
H7B	0.516843	0.042475	−0.026048	0.064*	
H7C	0.647534	0.045416	0.060229	0.064*	
C8	0.6704 (5)	0.1944 (2)	−0.1462 (4)	0.0953 (16)	
H8A	0.669255	0.229266	−0.148853	0.114*	
H8B	0.751950	0.183073	−0.125860	0.114*	
H8C	0.621237	0.181647	−0.212391	0.114*	
C9	0.4929 (4)	0.23236 (11)	0.1078 (3)	0.0564 (9)	
H9A	0.464229	0.216650	0.153714	0.068*	
H9B	0.558068	0.253924	0.145463	0.068*	
H9C	0.428204	0.250577	0.058072	0.068*	
C10	0.2575 (3)	0.10497 (11)	0.1661 (2)	0.0362 (6)	
C11	0.1633 (3)	0.13739 (12)	0.1595 (2)	0.0428 (7)	
C12	0.0481 (3)	0.12024 (13)	0.1414 (3)	0.0525 (8)	
H12	−0.011475	0.142360	0.138276	0.063*	
C13	0.0193 (3)	0.07129 (14)	0.1277 (2)	0.0531 (9)	
C14	0.1118 (3)	0.03930 (12)	0.1352 (2)	0.0490 (8)	
H14	0.094931	0.006227	0.126572	0.059*	
C15	0.2281 (3)	0.05497 (11)	0.1551 (2)	0.0390 (6)	
C16	0.1869 (3)	0.19137 (12)	0.1699 (3)	0.0605 (9)	
H16A	0.269829	0.197512	0.182150	0.073*	
H16B	0.135141	0.207348	0.108126	0.073*	
H16C	0.170640	0.203603	0.225913	0.073*	
C17	−0.1068 (3)	0.05407 (19)	0.1057 (3)	0.0798 (13)	
H17A	−0.110277	0.019351	0.098586	0.096*	
H17B	−0.128522	0.063189	0.160898	0.096*	
H17C	−0.162240	0.068660	0.043644	0.096*	
C18	0.3221 (3)	0.01757 (11)	0.1617 (3)	0.0517 (8)	
H18A	0.397928	0.033449	0.175720	0.062*	
H18B	0.331256	−0.005010	0.215374	0.062*	
H18C	0.297023	0.000460	0.098044	0.062*	
C19	0.0817 (4)	0.1129 (2)	−0.0945 (3)	0.0864 (15)	
H19A	0.073605	0.083645	−0.061181	0.104*	
H19B	0.052908	0.139998	−0.068566	0.104*	
H19C	0.035169	0.109988	−0.166632	0.104*	
C20	0.2658 (4)	0.07802 (15)	−0.1540 (3)	0.0816 (13)	
H20A	0.270385	0.046047	−0.125855	0.098*	
H20B	0.200305	0.079321	−0.220072	0.098*	
H20C	0.340315	0.085374	−0.160120	0.098*	
C21	0.2352 (4)	0.17798 (14)	−0.1387 (3)	0.0667 (10)	
H21A	0.221783	0.204870	−0.101793	0.080*	
H21B	0.310611	0.182363	−0.145271	0.080*	
H21C	0.170601	0.176310	−0.205223	0.080*	
P	0.24013 (8)	0.12219 (3)	−0.07038 (6)	0.0456 (2)	
Cu1	0.58712 (3)	0.12496 (2)	0.22033 (2)	0.03340 (13)	
Cu2	0.37473 (3)	0.12434 (2)	0.09729 (2)	0.03274 (13)	

### Atomic displacement parameters (Å^2^)

**Table:**

	*U*^11^	*U*^22^	*U*^33^	*U*^12^	*U*^13^	*U*^23^
C1	0.0323 (15)	0.0399 (15)	0.0349 (14)	−0.0001 (12)	0.0150 (12)	0.0030 (12)
C2	0.0351 (17)	0.0472 (16)	0.0426 (16)	−0.0017 (13)	0.0190 (14)	−0.0047 (13)
C3	0.050 (2)	0.073 (2)	0.0488 (19)	−0.0064 (17)	0.0318 (17)	−0.0115 (17)
C4	0.054 (2)	0.077 (2)	0.0462 (19)	−0.0117 (18)	0.0282 (17)	0.0040 (17)
C5	0.057 (2)	0.0540 (19)	0.053 (2)	−0.0120 (16)	0.0234 (17)	0.0129 (16)
C6	0.0404 (17)	0.0453 (16)	0.0400 (16)	−0.0012 (13)	0.0152 (14)	0.0047 (13)
C7	0.049 (2)	0.0495 (18)	0.072 (2)	−0.0015 (15)	0.0367 (18)	−0.0122 (16)
C8	0.106 (4)	0.126 (4)	0.078 (3)	−0.030 (3)	0.063 (3)	0.006 (3)
C9	0.073 (2)	0.0370 (16)	0.067 (2)	0.0041 (16)	0.0358 (19)	0.0051 (16)
C10	0.0283 (15)	0.0459 (16)	0.0339 (14)	−0.0003 (12)	0.0119 (12)	−0.0013 (12)
C11	0.0336 (16)	0.0562 (17)	0.0378 (16)	0.0050 (14)	0.0137 (13)	−0.0015 (14)
C12	0.0307 (17)	0.081 (2)	0.0451 (18)	0.0086 (16)	0.0150 (14)	−0.0009 (16)
C13	0.0315 (17)	0.086 (3)	0.0418 (18)	−0.0104 (16)	0.0144 (14)	−0.0045 (17)
C14	0.0411 (18)	0.063 (2)	0.0443 (17)	−0.0192 (16)	0.0184 (14)	−0.0052 (15)
C15	0.0355 (16)	0.0478 (16)	0.0348 (15)	−0.0065 (13)	0.0151 (13)	−0.0042 (13)
C16	0.046 (2)	0.057 (2)	0.078 (3)	0.0148 (17)	0.0246 (18)	−0.0009 (18)
C17	0.041 (2)	0.129 (4)	0.070 (3)	−0.025 (2)	0.023 (2)	−0.012 (2)
C18	0.052 (2)	0.0406 (16)	0.065 (2)	−0.0060 (14)	0.0261 (17)	−0.0058 (15)
C19	0.044 (2)	0.164 (5)	0.041 (2)	−0.016 (3)	0.0063 (17)	0.015 (2)
C20	0.076 (3)	0.081 (3)	0.066 (3)	0.007 (2)	0.005 (2)	−0.025 (2)
C21	0.072 (3)	0.073 (2)	0.054 (2)	0.013 (2)	0.024 (2)	0.0139 (18)
P	0.0392 (5)	0.0616 (5)	0.0320 (4)	−0.0034 (4)	0.0103 (3)	0.0025 (3)
Cu1	0.0285 (2)	0.0388 (2)	0.0322 (2)	0.00097 (13)	0.01154 (16)	0.00234 (13)
Cu2	0.0284 (2)	0.0384 (2)	0.0314 (2)	−0.00060 (13)	0.01196 (15)	0.00072 (13)

### Geometric parameters (Å, º)

**Table:**

C1—C2	1.415 (4)	C13—C14	1.392 (5)
C1—C6	1.414 (4)	C13—C17	1.502 (5)
C1—Cu1	2.006 (3)	C14—H14	0.9300
C1—Cu2	2.095 (3)	C14—C15	1.385 (4)
C2—C3	1.388 (4)	C15—C18	1.507 (4)
C2—C7	1.501 (4)	C16—H16A	0.9600
C3—H3	0.9300	C16—H16B	0.9600
C3—C4	1.377 (5)	C16—H16C	0.9600
C4—C5	1.385 (5)	C17—H17A	0.9600
C4—C8	1.516 (5)	C17—H17B	0.9600
C5—H5	0.9300	C17—H17C	0.9600
C5—C6	1.395 (4)	C18—H18A	0.9600
C6—C9	1.500 (4)	C18—H18B	0.9600
C7—H7A	0.9600	C18—H18C	0.9600
C7—H7B	0.9600	C19—H19A	0.9600
C7—H7C	0.9600	C19—H19B	0.9600
C8—H8A	0.9600	C19—H19C	0.9600
C8—H8B	0.9600	C19—P	1.820 (4)
C8—H8C	0.9600	C20—H20A	0.9600
C9—H9A	0.9600	C20—H20B	0.9600
C9—H9B	0.9600	C20—H20C	0.9600
C9—H9C	0.9600	C20—P	1.818 (4)
C10—C11	1.419 (4)	C21—H21A	0.9600
C10—C15	1.414 (4)	C21—H21B	0.9600
C10—Cu1^i^	2.005 (3)	C21—H21C	0.9600
C10—Cu2	2.093 (3)	C21—P	1.808 (4)
C11—C12	1.390 (5)	P—Cu2	2.2967 (9)
C11—C16	1.509 (5)	Cu1—Cu1^i^	2.5657 (7)
C12—H12	0.9300	Cu1—Cu2	2.4603 (5)
C12—C13	1.386 (5)	Cu1—Cu2^i^	2.4625 (5)
			
C2—C1—Cu1	110.9 (2)	C11—C16—H16B	109.5
C2—C1—Cu2	117.0 (2)	C11—C16—H16C	109.5
C6—C1—C2	116.7 (3)	H16A—C16—H16B	109.5
C6—C1—Cu1	116.0 (2)	H16A—C16—H16C	109.5
C6—C1—Cu2	115.2 (2)	H16B—C16—H16C	109.5
Cu1—C1—Cu2	73.70 (9)	C13—C17—H17A	109.5
C1—C2—C7	119.6 (3)	C13—C17—H17B	109.5
C3—C2—C1	120.9 (3)	C13—C17—H17C	109.5
C3—C2—C7	119.5 (3)	H17A—C17—H17B	109.5
C2—C3—H3	119.0	H17A—C17—H17C	109.5
C4—C3—C2	122.0 (3)	H17B—C17—H17C	109.5
C4—C3—H3	119.0	C15—C18—H18A	109.5
C3—C4—C5	117.9 (3)	C15—C18—H18B	109.5
C3—C4—C8	120.7 (4)	C15—C18—H18C	109.5
C5—C4—C8	121.4 (4)	H18A—C18—H18B	109.5
C4—C5—H5	119.1	H18A—C18—H18C	109.5
C4—C5—C6	121.8 (3)	H18B—C18—H18C	109.5
C6—C5—H5	119.1	H19A—C19—H19B	109.5
C1—C6—C9	120.7 (3)	H19A—C19—H19C	109.5
C5—C6—C1	120.7 (3)	H19B—C19—H19C	109.5
C5—C6—C9	118.6 (3)	P—C19—H19A	109.5
C2—C7—H7A	109.5	P—C19—H19B	109.5
C2—C7—H7B	109.5	P—C19—H19C	109.5
C2—C7—H7C	109.5	H20A—C20—H20B	109.5
H7A—C7—H7B	109.5	H20A—C20—H20C	109.5
H7A—C7—H7C	109.5	H20B—C20—H20C	109.5
H7B—C7—H7C	109.5	P—C20—H20A	109.5
C4—C8—H8A	109.5	P—C20—H20B	109.5
C4—C8—H8B	109.5	P—C20—H20C	109.5
C4—C8—H8C	109.5	H21A—C21—H21B	109.5
H8A—C8—H8B	109.5	H21A—C21—H21C	109.5
H8A—C8—H8C	109.5	H21B—C21—H21C	109.5
H8B—C8—H8C	109.5	P—C21—H21A	109.5
C6—C9—H9A	109.5	P—C21—H21B	109.5
C6—C9—H9B	109.5	P—C21—H21C	109.5
C6—C9—H9C	109.5	C19—P—Cu2	116.81 (13)
H9A—C9—H9B	109.5	C20—P—C19	103.0 (2)
H9A—C9—H9C	109.5	C20—P—Cu2	117.98 (14)
H9B—C9—H9C	109.5	C21—P—C19	102.3 (2)
C11—C10—Cu1^i^	110.2 (2)	C21—P—C20	100.9 (2)
C11—C10—Cu2	119.0 (2)	C21—P—Cu2	113.53 (14)
C15—C10—C11	116.5 (3)	C1—Cu1—Cu1^i^	110.85 (8)
C15—C10—Cu1^i^	117.9 (2)	C1—Cu1—Cu2	54.80 (8)
C15—C10—Cu2	112.5 (2)	C1—Cu1—Cu2^i^	161.89 (8)
Cu1^i^—C10—Cu2	73.85 (9)	C10^i^—Cu1—C1	138.26 (11)
C10—C11—C16	119.9 (3)	C10^i^—Cu1—Cu1^i^	110.89 (8)
C12—C11—C10	120.9 (3)	C10^i^—Cu1—Cu2^i^	54.72 (8)
C12—C11—C16	119.2 (3)	C10^i^—Cu1—Cu2	161.12 (8)
C11—C12—H12	118.9	Cu2^i^—Cu1—Cu1^i^	58.546 (15)
C13—C12—C11	122.2 (3)	Cu2—Cu1—Cu1^i^	58.630 (15)
C13—C12—H12	118.9	Cu2—Cu1—Cu2^i^	117.169 (16)
C12—C13—C14	117.1 (3)	C1—Cu2—P	97.94 (8)
C12—C13—C17	120.8 (4)	C1—Cu2—Cu1	51.50 (8)
C14—C13—C17	122.1 (4)	C1—Cu2—Cu1^i^	111.73 (8)
C13—C14—H14	118.8	C10—Cu2—C1	163.04 (11)
C15—C14—C13	122.3 (3)	C10—Cu2—P	99.02 (8)
C15—C14—H14	118.8	C10—Cu2—Cu1	111.87 (8)
C10—C15—C18	120.6 (3)	C10—Cu2—Cu1^i^	51.43 (8)
C14—C15—C10	121.0 (3)	P—Cu2—Cu1^i^	149.44 (3)
C14—C15—C18	118.4 (3)	P—Cu2—Cu1	147.71 (3)
C11—C16—H16A	109.5	Cu1—Cu2—Cu1^i^	62.824 (16)
			
C1—C2—C3—C4	1.5 (5)	C15—C10—C11—C12	−1.1 (4)
C2—C1—C6—C5	0.8 (4)	C15—C10—C11—C16	−179.7 (3)
C2—C1—C6—C9	178.6 (3)	C16—C11—C12—C13	177.8 (3)
C2—C3—C4—C5	−0.7 (6)	C17—C13—C14—C15	179.8 (3)
C2—C3—C4—C8	179.3 (4)	Cu1—C1—C2—C3	−137.4 (3)
C3—C4—C5—C6	−0.2 (5)	Cu1—C1—C2—C7	42.3 (3)
C4—C5—C6—C1	0.1 (5)	Cu1—C1—C6—C5	134.4 (3)
C4—C5—C6—C9	−177.8 (3)	Cu1—C1—C6—C9	−47.8 (4)
C6—C1—C2—C3	−1.6 (4)	Cu1^i^—C10—C11—C12	−139.0 (3)
C6—C1—C2—C7	178.2 (3)	Cu1^i^—C10—C11—C16	42.5 (3)
C7—C2—C3—C4	−178.2 (3)	Cu1^i^—C10—C15—C14	136.9 (3)
C8—C4—C5—C6	179.9 (4)	Cu1^i^—C10—C15—C18	−45.0 (4)
C10—C11—C12—C13	−0.7 (5)	Cu2—C1—C2—C3	140.8 (3)
C11—C10—C15—C14	2.3 (4)	Cu2—C1—C2—C7	−39.4 (4)
C11—C10—C15—C18	−179.6 (3)	Cu2—C1—C6—C5	−142.2 (3)
C11—C12—C13—C14	1.4 (5)	Cu2—C1—C6—C9	35.6 (4)
C11—C12—C13—C17	−178.5 (3)	Cu2—C10—C11—C12	138.7 (3)
C12—C13—C14—C15	−0.2 (5)	Cu2—C10—C11—C16	−39.8 (4)
C13—C14—C15—C10	−1.8 (5)	Cu2—C10—C15—C14	−140.1 (2)
C13—C14—C15—C18	−179.9 (3)	Cu2—C10—C15—C18	38.0 (3)

Symmetry code: (i) −*x*+1, *y*, −*z*+1/2.
